# Supplementary material for: A Critical View of the Application of the APEX Software (Aqueous Photochemistry of Environmentally-Occurring Xenobiotics) to Predict Photoreaction Kinetics in Surface Freshwaters
Source: Molecules. 2019 Dec 18;25(1):9. doi: 10.3390/molecules25010009 (PMC7017383; doi:10.3390/molecules25010009)
Supplement: Supplementary file 1 [file molecules-25-00009-s001.zip › 3.2.4_gcc-4.4.0.zip!/3.2.4_gcc-4.4.0/3.2.4_gcc-4.4.0/doc/octave/html/interpreter/Two_002dDimensional-Plots.html]

Two-Dimensional Plots - Untitled


Next: Three-Dimensional Plotting,
Up: Plotting Basics


---

#### 15.1.1 Two-Dimensional Plots

The `plot` function allows you to create simple x-y plots with
linear axes. For example,

```
     x = -10:0.1:10;
     plot (x, sin (x));
```

displays a sine wave shown in fig:plot. On most systems, this
command will open a separate plot window to display the graph.

**Figure 15.1: Simple Two-Dimensional Plot.**

— Function File: **plot** (y)  
— Function File: **plot** (x, y)  
— Function File: **plot** (x, y, property, value, ...)  
— Function File: **plot** (x, y, fmt)  
— Function File: **plot** (h, ...)  
> Produces two-dimensional plots. Many different combinations of
> arguments are possible. The simplest form is
>
> ```
>           plot (y)
> ```
>
> where the argument is taken as the set of y coordinates and the
> x coordinates are taken to be the indices of the elements,
> starting with 1.
>
> To save a plot, in one of several image formats such as PostScript
> or PNG, use the `print` command.
>
> If more than one argument is given, they are interpreted as
>
> ```
>           plot (y, property, value, ...)
> ```
>
> or
>
> ```
>           plot (x, y, property, value, ...)
> ```
>
> or
>
> ```
>           plot (x, y, fmt, ...)
> ```
>
> and so on. Any number of argument sets may appear. The x and
> y values are interpreted as follows:
>
> - If a single data argument is supplied, it is taken as the set of y
>   coordinates and the x coordinates are taken to be the indices of
>   the elements, starting with 1.- If the x is a vector and y is a matrix, then
>     the columns (or rows) of y are plotted versus x.
>     (using whichever combination matches, with columns tried first.)- If the x is a matrix and y is a vector,
>       y is plotted versus the columns (or rows) of x.
>       (using whichever combination matches, with columns tried first.)- If both arguments are vectors, the elements of y are plotted versus
>         the elements of x.- If both arguments are matrices, the columns of y are plotted
>           versus the columns of x. In this case, both matrices must have
>           the same number of rows and columns and no attempt is made to transpose
>           the arguments to make the number of rows match.
>
>           If both arguments are scalars, a single point is plotted.
>
> Multiple property-value pairs may be specified, but they must appear
> in pairs. These arguments are applied to the lines drawn by
> `plot`.
>
> If the fmt argument is supplied, it is interpreted as
> follows. If fmt is missing, the default gnuplot line style
> is assumed.
>
> -: Set lines plot style (default). .: Set dots plot style. n: Interpreted as the plot color if n is an integer in the range 1 to 6. nm: If nm is a two digit integer and m is an integer in the range 1 to 6, m is interpreted as the point style. This is only valid in combination with the `@` or `-@` specifiers. c: If c is one of `"k"` (black), `"r"` (red), `"g"` (green), `"b"` (blue), `"m"` (magenta), `"c"` (cyan), or `"w"` (white), it is interpreted as the line plot color. ";title;": Here `"title"` is the label for the key. + \* o x ^: Used in combination with the points or linespoints styles, set the point style.
>
> The fmt argument may also be used to assign key titles.
> To do so, include the desired title between semi-colons after the
> formatting sequence described above, e.g., "+3;Key Title;"
> Note that the last semi-colon is required and will generate an error if
> it is left out.
>
> Here are some plot examples:
>
> ```
>           plot (x, y, "@12", x, y2, x, y3, "4", x, y4, "+")
> ```
>
> This command will plot `y` with points of type 2 (displayed as
> +) and color 1 (red), `y2` with lines, `y3` with lines of
> color 4 (magenta) and `y4` with points displayed as +.
>
> ```
>           plot (b, "*", "markersize", 3)
> ```
>
> This command will plot the data in the variable `b`,
> with points displayed as \* with a marker size of 3.
>
> ```
>           t = 0:0.1:6.3;
>           plot (t, cos(t), "-;cos(t);", t, sin(t), "+3;sin(t);");
> ```
>
> This will plot the cosine and sine functions and label them accordingly
> in the key.
>
> If the first argument is an axis handle, then plot into these axes,
> rather than the current axis handle returned by `gca`.
>
>
>
> **See also:** semilogx, semilogy, loglog, polar, mesh, contour, bar, stairs, errorbar, xlabel, ylabel, title, print.

The `plotyy` function may be used to create a plot with two
independent y axes.

— Function File: **plotyy** (x1, y1, x2, y2)  
— Function File: **plotyy** (..., fun)  
— Function File: **plotyy** (..., fun1, fun2)  
— Function File: **plotyy** (h, ...)  
— Function File: [ax, h1, h2] = **plotyy** (...)  
> Plots two sets of data with independent y-axes. The arguments x1 and
> y1 define the arguments for the first plot and x1 and y2
> for the second.
>
> By default the arguments are evaluated with
> `feval (@plot,` x`,` y`)`. However the type of plot can be
> modified with the fun argument, in which case the plots are
> generated by `feval (`fun`,` x`,` y`)`. fun can be
> a function handle, an inline function or a string of a function name.
>
> The function to use for each of the plots can be independently defined
> with fun1 and fun2.
>
> If given, h defines the principal axis in which to plot the x1
> and y1 data. The return value ax is a two element vector with
> the axis handles of the two plots. h1 and h2 are handles to
> the objects generated by the plot commands.
>
> ```
>           x = 0:0.1:2*pi;
>           y1 = sin (x);
>           y2 = exp (x - 1);
>           ax = plotyy (x, y1, x - 1, y2, @plot, @semilogy);
>           xlabel ("X");
>           ylabel (ax(1), "Axis 1");
>           ylabel (ax(2), "Axis 2");
> ```

The functions `semilogx`, `semilogy`, and `loglog` are
similar to the `plot` function, but produce plots in which one or
both of the axes use log scales.

— Function File: **semilogx** (args)  
> Produce a two-dimensional plot using a log scale for the x
> axis. See the description of `plot` for a description of the
> arguments that `semilogx` will accept.
>
>
>
> **See also:** plot, semilogy, loglog.

— Function File: **semilogy** (args)  
> Produce a two-dimensional plot using a log scale for the y
> axis. See the description of `plot` for a description of the
> arguments that `semilogy` will accept.
>
>
>
> **See also:** plot, semilogx, loglog.

— Function File: **loglog** (args)  
> Produce a two-dimensional plot using log scales for both axes. See
> the description of `plot` for a description of the arguments
> that `loglog` will accept.
>
>
>
> **See also:** plot, semilogx, semilogy.

The functions `bar`, `barh`, `stairs`, and `stem`
are useful for displaying discrete data. For example,

```
     hist (randn (10000, 1), 30);
```

produces the histogram of 10,000 normally distributed random numbers
shown in fig:hist.

**Figure 15.2: Histogram.**

— Function File: **bar** (x, y)  
— Function File: **bar** (y)  
— Function File: **bar** (x, y, w)  
— Function File: **bar** (x, y, w, style)  
— Function File: h = **bar** (..., prop, val)  
— Function File: **bar** (h, ...)  
> Produce a bar graph from two vectors of x-y data.
>
> If only one argument is given, it is taken as a vector of y-values
> and the x coordinates are taken to be the indices of the elements.
>
> The default width of 0.8 for the bars can be changed using w.
>
> If y is a matrix, then each column of y is taken to be a
> separate bar graph plotted on the same graph. By default the columns
> are plotted side-by-side. This behavior can be changed by the style
> argument, which can take the values `"grouped"` (the default),
> or `"stacked"`.
>
> The optional return value h provides a handle to the "bar series"
> object with one handle per column of the variable y. This
> series allows common elements of the group of bar series objects to
> be changed in a single bar series and the same properties are changed
> in the other "bar series". For example
>
> ```
>           h = bar (rand (5, 10));
>           set (h(1), "basevalue", 0.5);
> ```
>
> changes the position on the base of all of the bar series.
>
> The optional input handle h allows an axis handle to be passed.
> Properties of the patch graphics object can be changed using
> prop, val pairs.
>
>
>
> **See also:** barh, plot.

— Function File: **barh** (x, y)  
— Function File: **barh** (y)  
— Function File: **barh** (x, y, w)  
— Function File: **barh** (x, y, w, style)  
— Function File: h = **barh** (..., prop, val)  
— Function File: **barh** (h, ...)  
> Produce a horizontal bar graph from two vectors of x-y data.
>
> If only one argument is given, it is taken as a vector of y-values
> and the x coordinates are taken to be the indices of the elements.
>
> The default width of 0.8 for the bars can be changed using w.
>
> If y is a matrix, then each column of y is taken to be a
> separate bar graph plotted on the same graph. By default the columns
> are plotted side-by-side. This behavior can be changed by the style
> argument, which can take the values `"grouped"` (the default),
> or `"stacked"`.
>
> The optional return value h provides a handle to the bar series
> object. See `bar` for a description of the use of the bar series.
>
> The optional input handle h allows an axis handle to be passed.
> Properties of the patch graphics object can be changed using
> prop, val pairs.
>
>
>
> **See also:** bar, plot.

— Function File: **hist** (y, x, norm)  
> Produce histogram counts or plots.
>
> With one vector input argument, plot a histogram of the values with
> 10 bins. The range of the histogram bins is determined by the range
> of the data. With one matrix input argument, plot a histogram where
> each bin contains a bar per input column.
>
> Given a second scalar argument, use that as the number of bins.
>
> Given a second vector argument, use that as the centers of the bins,
> with the width of the bins determined from the adjacent values in
> the vector.
>
> If third argument is provided, the histogram is normalized such that
> the sum of the bars is equal to norm.
>
> Extreme values are lumped in the first and last bins.
>
> With two output arguments, produce the values nn and xx such
> that `bar (`xx`,` nn`)` will plot the histogram.
>
>
>
> **See also:** bar.

— Function File: **stairs** (x, y)  
— Function File: **stairs** (..., style)  
— Function File: **stairs** (..., prop, val)  
— Function File: **stairs** (h, ...)  
— Function File: h = **stairs** (...)  
> Produce a stairstep plot. The arguments may be vectors or matrices.
>
> If only one argument is given, it is taken as a vector of y-values
> and the x coordinates are taken to be the indices of the elements.
>
> If two output arguments are specified, the data are generated but
> not plotted. For example,
>
> ```
>           stairs (x, y);
> ```
>
> and
>
> ```
>           [xs, ys] = stairs (x, y);
>           plot (xs, ys);
> ```
>
> are equivalent.
>
>
>
> **See also:** plot, semilogx, semilogy, loglog, polar, mesh, contour, bar, xlabel, ylabel, title.

— Function File: h = **stem** (x, y, linespec)  
— Function File: h = **stem** (..., "filled")  
> Plot a stem graph from two vectors of x-y data. If only one argument
> is given, it is taken as the y-values and the x coordinates are taken
> from the indices of the elements.
>
> If y is a matrix, then each column of the matrix is plotted as
> a separate stem graph. In this case x can either be a vector,
> the same length as the number of rows in y, or it can be a
> matrix of the same size as y.
>
> The default color is `"r"` (red). The default line style is
> `"-"` and the default marker is `"o"`. The line style can
> be altered by the `linespec` argument in the same manner as the
> `plot` command. For example
>
> ```
>           x = 1:10;
>           y = ones (1, length (x))*2.*x;
>           stem (x, y, "b");
> ```
>
> plots 10 stems with heights from 2 to 20 in blue;
>
> The return value of `stem` is a vector if "stem series" graphics
> handles, with one handle per column of the variable y. This
> handle regroups the elements of the stem graph together as the
> children of the "stem series" handle, allowing them to be altered
> together. For example
>
> ```
>           x = [0 : 10].';
>           y = [sin(x), cos(x)]
>           h = stem (x, y);
>           set (h(2), "color", "g");
>           set (h(1), "basevalue", -1)
> ```
>
> changes the color of the second "stem series" and moves the base line
> of the first.
>
>
>
> **See also:** bar, barh, plot.

— Function File: h = **stem3** (x, y, z, linespec)  
> Plot a three-dimensional stem graph and return the handles of the line
> and marker objects used to draw the stems as "stem series" object.
> The default color is `"r"` (red). The default line style is
> `"-"` and the default marker is `"o"`.
>
> For example,
>
> ```
>           theta = 0:0.2:6;
>           stem3 (cos (theta), sin (theta), theta)
> ```
>
> plots 31 stems with heights from 0 to 6 lying on a circle. Color
> definitions with rgb-triples are not valid!
>
>
>
> **See also:** bar, barh, stem, plot.

— Function File: **scatter** (x, y, s, c)  
— Function File: **scatter** (..., 'filled')  
— Function File: **scatter** (..., style)  
— Function File: **scatter** (..., prop, val)  
— Function File: **scatter** (h, ...)  
— Function File: h = **scatter** (...)  
> Plot a scatter plot of the data. A marker is plotted at each point
> defined by the points in the vectors x and y. The size of
> the markers used is determined by the s, which can be a scalar,
> a vector of the same length of x and y. If s is not
> given or is an empty matrix, then the default value of 8 points is used.
>
> The color of the markers is determined by c, which can be a string
> defining a fixed color, a 3 element vector giving the red, green and blue
> components of the color, a vector of the same length as x that gives
> a scaled index into the current colormap, or a n-by-3 matrix defining
> the colors of each of the markers individually.
>
> The marker to use can be changed with the style argument, that is a
> string defining a marker in the same manner as the `plot` command.
> If the argument 'filled' is given then the markers as filled. All
> additional arguments are passed to the underlying patch command.
>
> The optional return value h provides a handle to the patch object
>
> ```
>           x = randn (100, 1);
>           y = randn (100, 1);
>           scatter (x, y, [], sqrt(x.^2 + y.^2));
> ```
>
>
>
>
> **See also:** plot, patch, scatter3.

— Function File: **scatter3** (x, y, z, s, c)  
— Function File: **scatter3** (..., 'filled')  
— Function File: **scatter3** (..., style)  
— Function File: **scatter3** (..., prop, val)  
— Function File: **scatter3** (h, ...)  
— Function File: h = **scatter3** (...)  
> Plot a scatter plot of the data in 3D. A marker is plotted at each point
> defined by the points in the vectors x, y and z. The size
> of the markers used is determined by s, which can be a scalar or
> a vector of the same length of x, y and z. If s is
> not given or is an empty matrix, then the default value of 8 points is used.
>
> The color of the markers is determined by c, which can be a string
> defining a fixed color, a 3 element vector giving the red, green and blue
> components of the color, a vector of the same length as x that gives
> a scaled index into the current colormap, or a n-by-3 matrix defining
> the colors of each of the markers individually.
>
> The marker to use can be changed with the style argument, that is a
> string defining a marker in the same manner as the `plot` command.
> If the argument 'filled' is given then the markers as filled. All
> additional arguments are passed to the underlying patch command.
>
> The optional return value h provides a handle to the patch object
>
> ```
>           [x, y, z] = peaks (20);
>           scatter3 (x(:), y(:), z(:), [], z(:));
> ```
>
>
>
>
> **See also:** plot, patch, scatter.

— Function File: **plotmatrix** (x, y)  
— Function File: **plotmatrix** (x)  
— Function File: **plotmatrix** (..., style)  
— Function File: **plotmatrix** (h, ...)  
— Function File: [h, ax, bigax, p, pax] = **plotmatrix** (...)  
> Scatter plot of the columns of one matrix against another. Given the
> arguments x and y, that have a matching number of rows,
> `plotmatrix` plots a set of axes corresponding to
>
> ```
>           plot (x (:, i), y (:, j)
> ```
>
> Given a single argument x, then this is equivalent to
>
> ```
>           plotmatrix (x, x)
> ```
>
> except that the diagonal of the set of axes will be replaced with the
> histogram `hist (`x `(:, i))`.
>
> The marker to use can be changed with the style argument, that is a
> string defining a marker in the same manner as the `plot`
> command. If a leading axes handle h is passed to
> `plotmatrix`, then this axis will be used for the plot.
>
> The optional return value h provides handles to the individual
> graphics objects in the scatter plots, whereas ax returns the
> handles to the scatter plot axis objects. bigax is a hidden
> axis object that surrounds the other axes, such that the commands
> `xlabel`, `title`, etc., will be associated with this hidden
> axis. Finally p returns the graphics objects associated with
> the histogram and pax the corresponding axes objects.
>
> ```
>           plotmatrix (randn (100, 3), 'g+')
> ```

— Function File: **pareto** (x)  
— Function File: **pareto** (x, y)  
— Function File: **pareto** (h, ...)  
— Function File: h = **pareto** (...)  
> Draw a Pareto chart, also called ABC chart. A Pareto chart is a bar graph
> used to arrange information in such a way that priorities for process
> improvement can be established. It organizes and displays information
> to show the relative importance of data. The chart is similar to the
> histogram or bar chart, except that the bars are arranged in decreasing
> order from left to right along the abscissa.
>
> The fundamental idea (Pareto principle) behind the use of Pareto
> diagrams is that the majority of an effect is due to a small subset of the
> causes, so for quality improvement the first few (as presented on the
> diagram) contributing causes to a problem usually account for the majority
> of the result. Thus, targeting these "major causes" for elimination
> results in the most cost-effective improvement scheme.
>
> The data are passed as x and the abscissa as y. If y is
> absent, then the abscissa are assumed to be `1 : length (`x`)`.
> y can be a string array, a cell array of strings or a numerical
> vector.
>
> An example of the use of `pareto` is
>
> ```
>           Cheese = {"Cheddar", "Swiss", "Camembert", ...
>                     "Munster", "Stilton", "Blue"};
>           Sold = [105, 30, 70, 10, 15, 20];
>           pareto(Sold, Cheese);
> ```

— Function File: **rose** (th, r)  
— Function File: **rose** (h, ...)  
— Function File: h = **rose** (...)  
— Function File: [r, th] = **rose** (...)  
> Plot an angular histogram. With one vector argument th, plots the
> histogram with 20 angular bins. If th is a matrix, then each column
> of th produces a separate histogram.
>
> If r is given and is a scalar, then the histogram is produced with
> r bins. If r is a vector, then the center of each bin are
> defined by the values of r.
>
> The optional return value h provides a list of handles to the
> the parts of the vector field (body, arrow and marker).
>
> If two output arguments are requested, then rather than plotting the
> histogram, the polar vectors necessary to plot the histogram are
> returned.
>
> ```
>           [r, t] = rose ([2*randn(1e5,1), pi + 2 * randn(1e5,1)]);
>           polar (r, t);
> ```
>
>
>
>
> **See also:** plot, compass, polar, hist.

The `contour`, `contourf` and `contourc` functions
produce two-dimensional contour plots from three-dimensional data.

— Function File: **contour** (z)  
— Function File: **contour** (z, vn)  
— Function File: **contour** (x, y, z)  
— Function File: **contour** (x, y, z, vn)  
— Function File: **contour** (..., style)  
— Function File: **contour** (h, ...)  
— Function File: [c, h] = **contour** (...)  
> Plot level curves (contour lines) of the matrix z, using the
> contour matrix c computed by `contourc` from the same
> arguments; see the latter for their interpretation. The set of
> contour levels, c, is only returned if requested. For example:
>
> ```
>           x = 0:2;
>           y = x;
>           z = x' * y;
>           contour (x, y, z, 2:3)
> ```
>
> The style to use for the plot can be defined with a line style style
> in a similar manner to the line styles used with the `plot` command.
> Any markers defined by style are ignored.
>
> The optional input and output argument h allows an axis handle to
> be passed to `contour` and the handles to the contour objects to be
> returned.
>
>
>
> **See also:** contourc, patch, plot.

— Function File: [c, h] = **contourf** (x, y, z, lvl)  
— Function File: [c, h] = **contourf** (x, y, z, n)  
— Function File: [c, h] = **contourf** (x, y, z)  
— Function File: [c, h] = **contourf** (z, n)  
— Function File: [c, h] = **contourf** (z, lvl)  
— Function File: [c, h] = **contourf** (z)  
— Function File: [c, h] = **contourf** (ax, ...)  
— Function File: [c, h] = **contourf** (..., "property", val)  
> Compute and plot filled contours of the matrix z.
> Parameters x, y and n or lvl are optional.
>
> The return value c is a 2xn matrix containing the contour lines
> as described in the help to the contourc function.
>
> The return value h is handle-vector to the patch objects creating
> the filled contours.
>
> If x and y are omitted they are taken as the row/column
> index of z. n is a scalar denoting the number of lines
> to compute. Alternatively lvl is a vector containing the
> contour levels. If only one value (e.g., lvl0) is wanted, set
> lvl to [lvl0, lvl0]. If both n or lvl are omitted
> a default value of 10 contour level is assumed.
>
> If provided, the filled contours are added to the axes object
> ax instead of the current axis.
>
> The following example plots filled contours of the `peaks`
> function.
>
> ```
>           [x, y, z] = peaks (50);
>           contourf (x, y, z, -7:9)
> ```
>
>
>
>
> **See also:** contour, contourc, patch.

— Function File: [c, lev] = **contourc** (x, y, z, vn)  
> Compute isolines (contour lines) of the matrix z.
> Parameters x, y and vn are optional.
>
> The return value lev is a vector of the contour levels.
> The return value c is a 2 by n matrix containing the
> contour lines in the following format
>
> ```
>           c = [lev1, x1, x2, ..., levn, x1, x2, ...
>                len1, y1, y2, ..., lenn, y1, y2, ...]
> ```
>
> in which contour line n has a level (height) of levn and
> length of lenn.
>
> If x and y are omitted they are taken as the row/column
> index of z. vn is either a scalar denoting the number of lines
> to compute or a vector containing the values of the lines. If only one
> value is wanted, set vn `= [val, val]`;
> If vn is omitted it defaults to 10.
>
> For example,
>
> ```
>           x = 0:2;
>           y = x;
>           z = x' * y;
>           contourc (x, y, z, 2:3)
>                =>   2.0000   2.0000   1.0000   3.0000   1.5000   2.0000
>                2.0000   1.0000   2.0000   2.0000   2.0000   1.5000
> ```
>
>
>
>
> **See also:** contour.

— Function File: **contour3** (z)  
— Function File: **contour3** (z, vn)  
— Function File: **contour3** (x, y, z)  
— Function File: **contour3** (x, y, z, vn)  
— Function File: **contour3** (..., style)  
— Function File: **contour3** (h, ...)  
— Function File: [c, h] = **contour3** (...)  
> Plot level curves (contour lines) of the matrix z, using the
> contour matrix c computed by `contourc` from the same
> arguments; see the latter for their interpretation. The contours are
> plotted at the Z level corresponding to their contour. The set of
> contour levels, c, is only returned if requested. For example:
>
> ```
>           contour3 (peaks (19));
>           hold on
>           surface (peaks (19), "facecolor", "none", "EdgeColor", "black")
>           colormap hot
> ```
>
> The style to use for the plot can be defined with a line style style
> in a similar manner to the line styles used with the `plot` command.
> Any markers defined by style are ignored.
>
> The optional input and output argument h allows an axis handle to
> be passed to `contour` and the handles to the contour objects to be
> returned.
>
>
>
> **See also:** contourc, patch, plot.

The `errorbar`, `semilogxerr`, `semilogyerr`, and
`loglogerr` functions produce plots with error bar markers. For
example,

```
     x = 0:0.1:10;
     y = sin (x);
     yp =  0.1 .* randn (size (x));
     ym = -0.1 .* randn (size (x));
     errorbar (x, sin (x), ym, yp);
```

produces the figure shown in fig:errorbar.

**Figure 15.3: Errorbar plot.**

— Function File: **errorbar** (args)  
> This function produces two-dimensional plots with errorbars. Many
> different combinations of arguments are possible. The simplest form is
>
> ```
>           errorbar (y, ey)
> ```
>
> where the first argument is taken as the set of y coordinates
> and the second argument ey is taken as the errors of the
> y values. x coordinates are taken to be the indices
> of the elements, starting with 1.
>
> If more than two arguments are given, they are interpreted as
>
> ```
>           errorbar (x, y, ..., fmt, ...)
> ```
>
> where after x and y there can be up to four error
> parameters such as ey, ex, ly, uy, etc.,
> depending on the plot type. Any number of argument sets may appear,
> as long as they are separated with a format string fmt.
>
> If y is a matrix, x and error parameters must also be matrices
> having same dimensions. The columns of y are plotted versus the
> corresponding columns of x and errorbars are drawn from
> the corresponding columns of error parameters.
>
> If fmt is missing, yerrorbars ("~") plot style is assumed.
>
> If the fmt argument is supplied, it is interpreted as in
> normal plots. In addition the following plot styles are supported by
> errorbar:
>
> ~: Set yerrorbars plot style (default). >: Set xerrorbars plot style. ~>: Set xyerrorbars plot style. #: Set boxes plot style. #~: Set boxerrorbars plot style. #~>: Set boxxyerrorbars plot style.
>
> Examples:
>
> ```
>           errorbar (x, y, ex, ">")
> ```
>
> produces an xerrorbar plot of y versus x with x
> errorbars drawn from x-ex to x+ex.
>
> ```
>           errorbar (x, y1, ey, "~",
>                     x, y2, ly, uy)
> ```
>
> produces yerrorbar plots with y1 and y2 versus x.
> Errorbars for y1 are drawn from y1-ey to
> y1+ey, errorbars for y2 from y2-ly to
> y2+uy.
>
> ```
>           errorbar (x, y, lx, ux,
>                     ly, uy, "~>")
> ```
>
> produces an xyerrorbar plot of y versus x in which
> x errorbars are drawn from x-lx to x+ux
> and y errorbars from y-ly to y+uy.
>
>
>
> **See also:** semilogxerr, semilogyerr, loglogerr.

— Function File: **semilogxerr** (args)  
> Produce two-dimensional plots on a semilogarithm axis with errorbars.
> Many different combinations of arguments are possible. The most used
> form is
>
> ```
>           semilogxerr (x, y, ey, fmt)
> ```
>
> which produces a semi-logarithm plot of y versus x
> with errors in the y-scale defined by ey and the plot
> format defined by fmt. See errorbar for available formats and
> additional information.
>
>
>
> **See also:** errorbar, loglogerr, semilogyerr.

— Function File: **semilogyerr** (args)  
> Produce two-dimensional plots on a semilogarithm axis with errorbars.
> Many different combinations of arguments are possible. The most used
> form is
>
> ```
>           semilogyerr (x, y, ey, fmt)
> ```
>
> which produces a semi-logarithm plot of y versus x
> with errors in the y-scale defined by ey and the plot
> format defined by fmt. See errorbar for available formats and
> additional information.
>
>
>
> **See also:** errorbar, loglogerr, semilogxerr.

— Function File: **loglogerr** (args)  
> Produce two-dimensional plots on double logarithm axis with
> errorbars. Many different combinations of arguments are possible.
> The most used form is
>
> ```
>           loglogerr (x, y, ey, fmt)
> ```
>
> which produces a double logarithm plot of y versus x
> with errors in the y-scale defined by ey and the plot
> format defined by fmt. See errorbar for available formats and
> additional information.
>
>
>
> **See also:** errorbar, semilogxerr, semilogyerr.

Finally, the `polar` function allows you to easily plot data in
polar coordinates. However, the display coordinates remain rectangular
and linear. For example,

```
     polar (0:0.1:10*pi, 0:0.1:10*pi);
```

produces the spiral plot shown in fig:polar.

**Figure 15.4: Polar plot.**

— Function File: **polar** (theta, rho, fmt)  
> Make a two-dimensional plot given the polar coordinates theta and
> rho.
>
> The optional third argument specifies the line type.
>
>
>
> **See also:** plot.

— Function File: **pie** (y)  
— Function File: **pie** (y, explode)  
— Function File: **pie** (..., labels)  
— Function File: **pie** (h, ...);  
— Function File: h = **pie** (...);  
> Produce a pie chart.
>
> Called with a single vector argument, produces a pie chart of the
> elements in x, with the size of the slice determined by percentage
> size of the values of x.
>
> The variable explode is a vector of the same length as x that
> if non zero 'explodes' the slice from the pie chart.
>
> If given labels is a cell array of strings of the same length as
> x, giving the labels of each of the slices of the pie chart.
>
> The optional return value h provides a handle to the patch object.
>
>
>
> **See also:** bar, stem.

— Function File: **quiver** (u, v)  
— Function File: **quiver** (x, y, u, v)  
— Function File: **quiver** (..., s)  
— Function File: **quiver** (..., style)  
— Function File: **quiver** (..., 'filled')  
— Function File: **quiver** (h, ...)  
— Function File: h = **quiver** (...)  
> Plot the `(`u`,` v`)` components of a vector field in
> an `(`x`,` y`)` meshgrid. If the grid is uniform, you can
> specify x and y as vectors.
>
> If x and y are undefined they are assumed to be
> `(1:`m`, 1:`n`)` where `[`m`,` n`] =
> size(`u`)`.
>
> The variable s is a scalar defining a scaling factor to use for
> the arrows of the field relative to the mesh spacing. A value of 0
> disables all scaling. The default value is 1.
>
> The style to use for the plot can be defined with a line style style
> in a similar manner to the line styles used with the `plot` command.
> If a marker is specified then markers at the grid points of the vectors are
> printed rather than arrows. If the argument 'filled' is given then the
> markers as filled.
>
> The optional return value h provides a quiver group that
> regroups the components of the quiver plot (body, arrow and marker),
> and allows them to be changed together
>
> ```
>           [x, y] = meshgrid (1:2:20);
>           h = quiver (x, y, sin (2*pi*x/10), sin (2*pi*y/10));
>           set (h, "maxheadsize", 0.33);
> ```
>
>
>
>
> **See also:** plot.

— Function File: **quiver3** (u, v, w)  
— Function File: **quiver3** (x, y, z, u, v, w)  
— Function File: **quiver3** (..., s)  
— Function File: **quiver3** (..., style)  
— Function File: **quiver3** (..., 'filled')  
— Function File: **quiver3** (h, ...)  
— Function File: h = **quiver3** (...)  
> Plot the `(`u`,` v`,` w`)` components of a vector field in
> an `(`x`,` y`),` z meshgrid. If the grid is uniform, you
> can specify x, y z as vectors.
>
> If x, y and z are undefined they are assumed to be
> `(1:`m`, 1:`n`, 1:`p`)` where `[`m`,` n`] =
> size(`u`)` and p `= max (size (`w`))`.
>
> The variable s is a scalar defining a scaling factor to use for
> the arrows of the field relative to the mesh spacing. A value of 0
> disables all scaling. The default value is 1.
>
> The style to use for the plot can be defined with a line style style
> in a similar manner to the line styles used with the `plot` command.
> If a marker is specified then markers at the grid points of the vectors are
> printed rather than arrows. If the argument 'filled' is given then the
> markers as filled.
>
> The optional return value h provides a quiver group that
> regroups the components of the quiver plot (body, arrow and marker),
> and allows them to be changed together
>
> ```
>           [x, y, z] = peaks (25);
>           surf (x, y, z);
>           hold on;
>           [u, v, w] = surfnorm (x, y, z / 10);
>           h = quiver3 (x, y, z, u, v, w);
>           set (h, "maxheadsize", 0.33);
> ```
>
>
>
>
> **See also:** plot.

— Function File: **compass** (u, v)  
— Function File: **compass** (z)  
— Function File: **compass** (..., style)  
— Function File: **compass** (h, ...)  
— Function File: h = **compass** (...)  
> Plot the `(`u`,` v`)` components of a vector field emanating
> from the origin of a polar plot. If a single complex argument z is
> given, then u `= real (`z`)` and v `= imag
> (`z`)`.
>
> The style to use for the plot can be defined with a line style style
> in a similar manner to the line styles used with the `plot` command.
>
> The optional return value h provides a list of handles to the
> the parts of the vector field (body, arrow and marker).
>
> ```
>           a = toeplitz([1;randn(9,1)],[1,randn(1,9)]);
>           compass (eig (a))
> ```
>
>
>
>
> **See also:** plot, polar, quiver, feather.

— Function File: **feather** (u, v)  
— Function File: **feather** (z)  
— Function File: **feather** (..., style)  
— Function File: **feather** (h, ...)  
— Function File: h = **feather** (...)  
> Plot the `(`u`,` v`)` components of a vector field emanating
> from equidistant points on the x-axis. If a single complex argument
> z is given, then u `= real (`z`)` and
> v `= imag (`z`)`.
>
> The style to use for the plot can be defined with a line style style
> in a similar manner to the line styles used with the `plot` command.
>
> The optional return value h provides a list of handles to the
> the parts of the vector field (body, arrow and marker).
>
> ```
>           phi = [0 : 15 : 360] * pi / 180;
>           feather (sin (phi), cos (phi))
> ```
>
>
>
>
> **See also:** plot, quiver, compass.

— Function File: **pcolor** (x, y, c)  
— Function File: **pcolor** (c)  
> Density plot for given matrices x, and y from `meshgrid` and
> a matrix c corresponding to the x and y coordinates of
> the mesh's vertices. If x and y are vectors, then a typical vertex
> is (x(j), y(i), c(i,j)). Thus, columns of c
> correspond to different x values and rows of c correspond
> to different y values.
>
> The `colormap` is scaled to the extents of c.
> Limits may be placed on the color axis by the
> command `caxis`, or by setting the `clim` property of the
> parent axis.
>
> The face color of each cell of the mesh is determined by interpolating
> the values of c for the cell's vertices. Contrast this with
> `imagesc` which renders one cell for each element of c.
>
> `shading` modifies an attribute determining the manner by which the
> face color of each cell is interpolated from the values of c,
> and the visibility of the cells' edges. By default the attribute is
> "faceted", which renders a single color for each cell's face with the edge
> visible.
>
> h is the handle to the surface object.
>
>
>
> **See also:** caxis, contour, meshgrid, imagesc, shading.

— Function File: **area** (x, y)  
— Function File: **area** (x, y, lvl)  
— Function File: **area** (..., prop, val, ...)  
— Function File: **area** (y, ...)  
— Function File: **area** (h, ...)  
— Function File: h = **area** (...)  
> Area plot of cumulative sum of the columns of y. This shows the
> contributions of a value to a sum, and is functionally similar to
> `plot (`x`, cumsum (`y`, 2))`, except that the area under
> the curve is shaded.
>
> If the x argument is omitted it is assumed to be given by
> `1 : rows (`y`)`. A value lvl can be defined that determines
> where the base level of the shading under the curve should be defined.
>
> Additional arguments to the `area` function are passed to the
> `patch`. The optional return value h provides a handle to
> area series object representing the patches of the areas.
>
>
>
> **See also:** plot, patch.

— Function File: **comet** (y)  
— Function File: **comet** (x, y)  
— Function File: **comet** (x, y, p)  
— Function File: **comet** (ax, ...)  
> Produce a simple comet style animation along the trajectory provided by
> the input coordinate vectors (x, y), where x will default
> to the indices of y.
>
> The speed of the comet may be controlled by p, which represents the
> time which passes as the animation passes from one point to the next. The
> default for p is 0.1 seconds.
>
> If ax is specified the animation is produced in that axis rather than
> the `gca`.

The axis function may be used to change the axis limits of an existing
plot and various other axis properties, such as the aspect ratio and the
appearance of tic marks.

— Function File: **axis** (limits)  
> Set axis limits for plots.
>
> The argument limits should be a 2, 4, or 6 element vector. The
> first and second elements specify the lower and upper limits for the x
> axis. The third and fourth specify the limits for the y-axis, and the
> fifth and sixth specify the limits for the z-axis.
>
> Without any arguments, `axis` turns autoscaling on.
>
> With one output argument, `x = axis` returns the current axes
>
> The vector argument specifying limits is optional, and additional
> string arguments may be used to specify various axis properties. For
> example,
>
> ```
>           axis ([1, 2, 3, 4], "square");
> ```
>
> forces a square aspect ratio, and
>
> ```
>           axis ("labely", "tic");
> ```
>
> turns tic marks on for all axes and tic mark labels on for the y-axis
> only.
>
> The following options control the aspect ratio of the axes.
>
> `"square"`: Force a square aspect ratio. `"equal"`: Force x distance to equal y-distance. `"normal"`: Restore the balance.
>
> The following options control the way axis limits are interpreted.
>
> `"auto"`: Set the specified axes to have nice limits around the data or all if no axes are specified. `"manual"`: Fix the current axes limits. `"tight"`: Fix axes to the limits of the data.
>
> The option `"image"` is equivalent to `"tight"` and
> `"equal"`.
>
> The following options affect the appearance of tic marks.
>
> `"on"`: Turn tic marks and labels on for all axes. `"off"`: Turn tic marks off for all axes. `"tic[xyz]"`: Turn tic marks on for all axes, or turn them on for the specified axes and off for the remainder. `"label[xyz]"`: Turn tic labels on for all axes, or turn them on for the specified axes and off for the remainder. `"nolabel"`: Turn tic labels off for all axes.
>
> Note, if there are no tic marks for an axis, there can be no labels.
>
> The following options affect the direction of increasing values on
> the axes.
>
> `"ij"`: Reverse y-axis, so lower values are nearer the top. `"xy"`: Restore y-axis, so higher values are nearer the top.
>
> If an axes handle is passed as the first argument, then operate on
> this axes rather than the current axes.

Similarly the axis limits of the colormap can be changed with the caxis
function.

— Function File: **caxis** (limits)  
— Function File: **caxis** (h, ...)  
> Set color axis limits for plots.
>
> The argument limits should be a 2 element vector specifying the
> lower and upper limits to assign to the first and last value in the
> colormap. Values outside this range are clamped to the first and last
> colormap entries.
>
> If limits is 'auto', then automatic colormap scaling is applied,
> whereas if limits is 'manual' the colormap scaling is set to manual.
>
> Called without any arguments to current color axis limits are returned.
>
> If an axes handle is passed as the first argument, then operate on
> this axes rather than the current axes.

The `xlim`, `ylim`, and `zlim` functions may be used to
get or set individual axis limits. Each has the same form.

— Function File: xl = **xlim** ()  
— Function File: **xlim** (xl)  
— Function File: m = **xlim** ('mode')  
— Function File: **xlim** (m)  
— Function File: **xlim** (h, ...)  
> Get or set the limits of the x-axis of the current plot. Called without
> arguments `xlim` returns the x-axis limits of the current plot.
> If passed a two element vector xl, the limits of the x-axis are set
> to this value.
>
> The current mode for calculation of the x-axis can be returned with a
> call `xlim ('mode')`, and can be either 'auto' or 'manual'. The
> current plotting mode can be set by passing either 'auto' or 'manual'
> as the argument.
>
> If passed an handle as the first argument, then operate on this handle
> rather than the current axes handle.
>
>
>
> **See also:** ylim, zlim, set, get, gca.

- Two-dimensional Function Plotting
